# Racial Equity in Living Donor Kidney Transplant Centers, 2008-2018

**DOI:** 10.1001/jamanetworkopen.2023.47826

**Published:** 2023-12-15

**Authors:** Lisa M. McElroy, Tyler Schappe, Dinushika Mohottige, LaShara Davis, Sarah B. Peskoe, Virginia Wang, Jane Pendergast, L. Ebony Boulware

**Affiliations:** 1 Department of Surgery, Duke University School of Medicine, Durham, North Carolina; 2 Department of Biostatistics and Bioinformatics, Duke University School of Medicine, Durham, North Carolina; 3 Institute of Health Equity Research and Barbara T. Murphy Division of Nephrology, Icahn School of Medicine at Mount Sinai, New York, New York; 4 Department of Surgery and J. C. Walter Jr Transplant Center, Houston Methodist Hospital, Houston, Texas; 5 Department of Population Health Sciences, Duke University School of Medicine, Durham, North Carolina; 6 Wake Forest University School of Medicine, Winston Salem, North Carolina

## Abstract

**Importance:**

It is unclear whether center-level factors are associated with racial equity in living donor kidney transplant (LDKT).

**Objective:**

To evaluate center-level factors and racial equity in LDKT during an 11-year time period.

**Design, Setting, and Participants:**

A retrospective cohort longitudinal study was completed in February 2023, of US transplant centers with at least 12 annual LDKTs from January 1, 2008, to December 31, 2018, identified in the Health Resources Services Administration database and linked to the US Renal Data System and the Scientific Registry of Transplant Recipients.

**Main Outcomes and Measures:**

Observed and model-based estimated Black-White mean LDKT rate ratios (RRs), where an RR of 1 indicates racial equity and values less than 1 indicate a lower rate of LDKT of Black patients compared with White patients. Estimated yearly best-case center-specific LDKT RRs between Black and White individuals, where modifiable center characteristics were set to values that would facilitate access to LDKT.

**Results:**

The final cohorts of patients included 394 625 waitlisted adults, of whom 33.1% were Black and 66.9% were White, and 57 222 adult LDKT recipients, of whom 14.1% were Black and 85.9% were White. Among 89 transplant centers, estimated yearly center-level RRs between Black and White individuals accounting for center and population characteristics ranged from 0.0557 in 2008 to 0.771 in 2018. The yearly median RRs ranged from 0.216 in 2016 to 0.285 in 2010. Model-based estimations for the hypothetical best-case scenario resulted in little change in the minimum RR (from 0.0557 to 0.0549), but a greater positive shift in the maximum RR from 0.771 to 0.895. Relative to the observed 582 LDKT in Black patients and 3837 in White patients, the 2018 hypothetical model estimated an increase of 423 (a 72.7% increase) LDKTs for Black patients and of 1838 (a 47.9% increase) LDKTs for White patients.

**Conclusions and Relevance:**

In this cohort study of patients with kidney failure, no substantial improvement occurred over time either in the observed or the covariate-adjusted estimated RRs. Under the best-case hypothetical estimations, modifying centers’ participation in the paired exchange and voucher programs and increased access to public insurance may contribute to improved racial equity in LDKT. Additional work is needed to identify center-level and program-specific strategies to improve racial equity in access to LDKT.

## Key Points

**Question:**

Do center-level racial disparities in living donor kidney transplant (LDKT) vary in light of differences in waiting list, referral region, and center characteristics?

**Findings:**

This cohort study of US Renal Data System and Scientific Registry of Transplant Recipients data on 57 222 adults who received LDKTs showed no improvement either in the observed or the covariate-adjusted LDKT rate ratios between Black and White individuals during the 11 years examined. Center-level modifiable factors showed potential for improving LDKT racial equity.

**Meaning:**

Additional work is necessary to identify transplant program and center-level strategies to improve racial equity in access to LDKT.

## Teaser

This cohort study examines racial equity among living donor kidney transplant recipients by the characteristics and catchment areas of transplant centers.

## Introduction

Kidney failure is most prevalent in Black individuals, and double the rate of new cases occur in Black compared with White patients.^1^ Living donor kidney transplant (LDKT) affords patients with kidney failure freedom from dialysis, improved quality of life, and excellent long-term graft survival.^2,3^ Racial disparities in LDKT have existed for decades, yet persist despite policy- and research-associated interventions.^4,5,6,7,8,9,10^

Health system and transplant center volume, referral population characteristics, and care delivery processes have been associated with a wide range of transplant care.^11,12,13,14,15,16,17^ Despite this, little work has been done to investigate how transplant center–specific characteristics and catchment area population characteristics might be associated with LDKT racial inequities.

Major efforts are needed to better understand the association between transplant centers and LDKT racial equity and whether center-level practice modifications may improve equity in LDKT rates. To that end, we examined racial differences in LDKT rates among US transplant centers in light of differences in waiting list, referral region, and center characteristics to identify targets for multilevel interventions.

## Methods

### Data Sources

We collected data on US kidney transplant (KT) centers using 3 national data sources: (1) the Health Resources Services Administration database of active US transplant centers,^18^ (2) the US Renal Data System,^19^ and (3) the Scientific Registry of Transplant Recipients. We defined geographic transplant referral regions (TRRs) for each transplant center based on hospital referral regions and locations of waitlisted patients (eFigure 7 in Supplement 1).^20^ We characterized TRRs using population demographic data sourced from the American Community Survey.^7,8^ The analysis was completed in February 2023. This study was deemed exempt from review and the requirement for informed consent by the Duke University Institutional Review Board based on use of a preexisting national data set. This study followed the Strengthening the Reporting of Observational Studies in Epidemiology (STROBE) reporting guideline.

### Study Period and Cohort Definitions

The study cohort consisted of US transplant centers that performed at least 12 LDKTs annually between 2008 and 2018. Data used to define this cohort were restricted to Black and White adults (age ≥18 years at time of transplant or during waitlisted period) who were waitlisted or underwent transplant between January 1, 2008, and December 31, 2018. The race variable from the US Renal Data System patients analysis file was used to determine patient race, which is reported by dialysis and transplant providers via the Centers for Medicaid & Medicare Services End-Stage Renal Disease Medical Evidence Report form 2728. Full cohort derivations are provided in eFigures 1-6 in Supplement 1.

### Outcome

The primary measure of interest was center yearly LDKT rate ratio (RR) between Black and White individuals. We calculated the rate of LDKT per eligible wait time for Black and White patients for each center in each year, then derived the ratio of those rates as the LDKT RR between Black and White individuals. An RR of 1 indicates equal rate (ie, racial equity) and lower than 1 indicates inequity for Black patients. The LDKT rate was defined as follows: LDKT rate*_j,k,t_* = LDKT events*_j,k,t_* / wait time*_j,k,t_*, where *j* = 1,2 race groups, *k* = 1…89 transplant centers, and *t* = 1…11 calendar years in the study period.

### Covariates

We derived modifiable and nonmodifiable covariates at 3 levels: (1) TRRs in which centers were located, (2) center characteristics, and (3) characteristics of patients who were waitlisted. Nonmodifiable covariates included characteristics of patients waitlisted for KT and TRR population. Characteristics of patients waitlisted included percentage female sex, calculated panel reactive antibody greater than 70%, less than postsecondary education, and type B blood. The TRR characteristics included Black population prevalence, percentage uninsured, and interquintile range of the Area Deprivation Index (ADI).^21,22^ Magnitude of variation in TRR-specific ADI was considered a proxy for socioeconomic inequality that may influence candidate access to the waiting list and the pool of eligible donors.

Modifiable covariates at the transplant center level included participation in the National Kidney Registry (NKR) voucher or a paired exchange program, state Medicaid expansion,^23,24,25^ and percentage of total KTs that were LDKT. Centers participating in either the NKR or paired exchange program as of 2023 were given credit for all years in the study period; nonparticipation was assumed for centers for which NKR voucher program status was unlisted. Medicaid expansion was used as a proxy for care to the uninsured.^26,27,28,29,30,31^ This was determined for each year based on expansion status of the state in which the center was located. Additional details on how covariates were sourced from raw data and derived can be found in the eMethods in Supplement 1.

### Statistical Analysis

We fit a generalized linear mixed-effects model that assumed a Poisson distribution and log link with the outcome of LDKT rates per center-year-race to achieve 3 objectives: (1) estimate center-specific yearly LDKT rates as a function of race, TRR characteristics, center-level KT waitlisted population, transplant center operational characteristics, and their interactions with race; (2) examine associations between observed covariates given in the above section and LDKT rates for each race; and (3) generate estimated LDKT RRs (with covariates as observed), while accounting for within-center dependence over time. The model included fixed effects for race and covariates and interactions with race that allowed for LDKT rates for Black and White patients to be estimated separately for each center and each year. We also included an offset term for wait time per center-year-race and random effects to capture dependence within centers over time. We checked for evidence of spatial dependence in model residuals using semivariograms.

Additionally, 2 sets of LDKT RR estimations corresponding to hypothetical scenarios were generated by modifying inputted covariate values but using the same regression coefficients. The first corresponded to a best-case scenario in which center-level modifiable covariates were fixed to values that facilitate transplant: all centers participated in NKR and paired exchange programs in all years for which the programs have existed, center percentage LDKT of total KT volume was fixed at the 90th percentile across all years, and the Affordable Care Act Medicaid expansion was applied to all states starting in 2014. The second was a set of risk-adjusted estimations that corresponded to an equalized scenario in which center-nonmodifiable covariates were fixed to the median observed values across all centers and years while modifiable covariates remained as observed.

Data analysis was performed using the R Project for Statistical Computing^32^ relevant packages^33,34,35,36,37,38,39^ and SAS software, version 9.4 (SAS Institute LLC). *P* values reported are 2-sided, with a significance threshold of .05.

## Results

**Figure 1.**
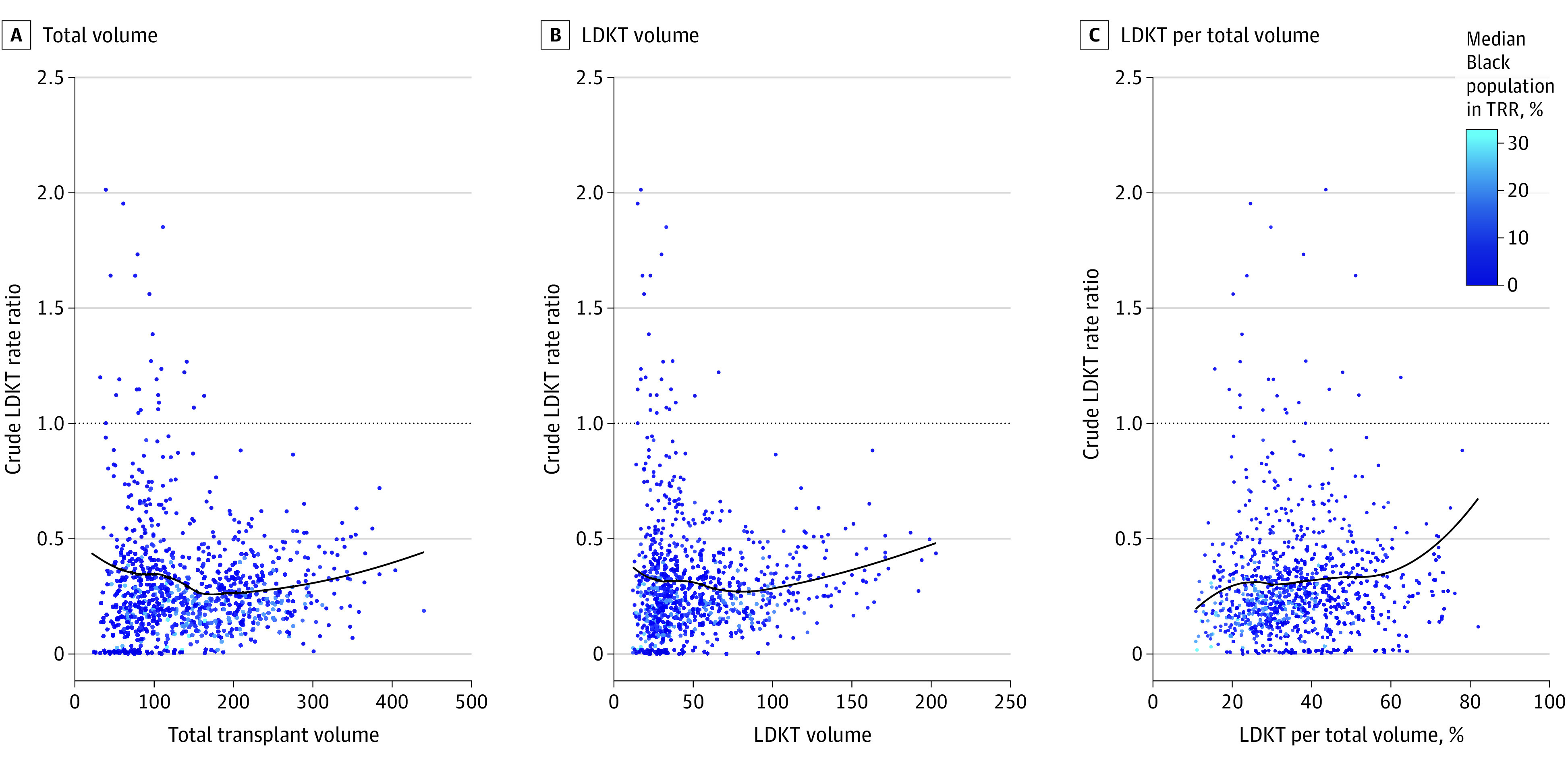
Observed Association Between Live Donor Kidney Transplant (LDKT) Black-White Patient Rate Ratio and Transplant Center Volume Total transplant volume (A), LDKT volume (B), and total percentage LDKT volume of total transplant volume (C). Blue color indicates the population-weighted median of percentage Black population among all census tracts or block groups in each center’s transplant referral region (TRR). Each data point represents a specific transplant center for a single year in the study period. Only centers that performed at least 12 LDKTs in all years of the study period are included. Data points with a y-axis value of 0 are jittered vertically to aid visualization. Although the y-axis reaches 2.5, the data points were truncated at 2.1 to aid in visualization, which excluded a single outlying point (center year) with a crude LDKT rate ratio of 4.27 from view. While the outlier data point affects the locally weighted scatterplot smoothing regression lines, no inferences or estimates are reported from this visualization, which does not inform the primary statistical analysis.

The final cohorts of patients used to derive the LDKT rate outcome included 394 625 adults who were waitlisted, of whom 33.1% were Black and 66.9% were White, and 57 222 adults who received LDKTs, of whom 14.1% were Black and 85.9% were White; there were no additional racial or ethnic categories included in this study. Summary statistics, including missing values, related to LDKTs performed and characteristics of transplant centers, patients who were waitlisted, and referral regions are presented for all study years in eTable 1 in Supplement 1. We observed an association between the prevalence of Black populations within a TRR and overall LDKT volume where low volume centers tend to have higher prevalence of Black populations (Figure 1). We saw a concave trend over time in the percentage of LDKTs that were United Network for Organ Sharing Kidney Paired Donation Program–matched runs (eTable 4 in Supplement 1). Further results are provided in the eResults in Supplement 1.

### LDKT RRs Between Black and White Individuals

Observed center-level LDKT RRs between Black and White individuals ranged from 0 to 4.27 during the study period, with yearly medians ranging from 0.197 (occurring in 2015) to 0.305 (occurring in 2010) (Table 1), indicating median lower rates of LDKT among Black compared with White patients. When patient, center, and regional characteristics were included in the estimation model, estimated center-level RRs over the entire study period ranged from 0.0577 to 0.771, and yearly medians of center RRs ranged from 0.216 (in 2016) to 0.285 (in 2010).

**Table 1.** Observed and Model-Estimated LDKT Black-White Rate Ratios^a^

Variable	2008 (n = 89)	2009 (n = 88)	2010 (n = 89)	2011 (n = 89)	2012 (n = 89)	2013 (n = 89)	2014 (n = 89)	2015 (n = 89)	2016 (n = 89)	2017 (n = 88)	2018 (n = 89)	Overall (n = 977)
**Observed LDKT Black-White rate ratio**												
Mean (SD)	0.363 (0.374)	0.274 (0.200)	0.357 (0.277)	0.288 (0.207)	0.282 (0.237)	0.274 (0.197)	0.284 (0.265)	0.238 (0.233)	0.278 (0.248)	0.305 (0.283)	0.337 (0.491)	0.298 (0.287)
Median (range)	0.298 (0-2.01)	0.232 (0-0.927)	0.305 (0-1.85)	0.252 (0-1.09)	0.243 (0-1.07)	0.241 (0-0.826)	0.255 (0-1.73)	0.197 (0-1.64)	0.217 (0-1.56)	0.239 (0-1.39)	0.231 (0-4.27)	0.246 (0-4.27)
**Estimated LDKT Black-White rate ratio**												
Mean (SD)	0.287 (0.131)	0.310 (0.132)	0.314 (0.126)	0.301 (0.116)	0.288 (0.110)	0.270 (0.102)	0.278 (0.111)	0.261 (0.107)	0.252 (0.106)	0.249 (0.109)	0.270 (0.118)	0.280 (0.117)
Median (range)	0.255 (0.0557-0.666)	0.278 (0.0925-0.692)	0.285 (0.0891-0.733)	0.268 (0.0735-0.718)	0.267 (0.120-0.732)	0.248 (0.0872-0.642)	0.254 (0.109-0.625)	0.232 (0.108-0.660)	0.216 (0.0973-0.698)	0.220 (0.0689-0.691)	0.246 (0.0659-0.771)	0.252 (0.0557-0.771)

Abbreviation: LDKT, living donor kidney transplant.

^a^ Estimated LDKT rate ratios are adjusted for percentage of waitlisted patients with calculated panel-reactive antibody greater than 0.7, percentage with type B blood, percentage with some postsecondary education attained, percentage female (patient-level), percentage LDKT of total transplant volume, participation in the National Kidney Registry voucher and in the paired exchange program, state Medicaid expansion (center-level), and percentage Black, percentage uninsured, and Area Deprivation Index in the transplant referral region level. Missing values refer to the study years with n = 88 centers (2009, 2017) in which 1 center in each year had an undefined rate ratio for the reasons stated. The missing value among the estimated LDKT rate ratio is due to a single center having an undefined rate ratio, and in 2009 is due to all patients waiting at a center during 2009 having missing values for the postsecondary education variable.

### Covariates in the Estimation Model

Across all centers, all years in the study period, and all levels of categorical covariates, with numeric covariates fixed at observed mean values, the estimated average LDKT RR between Black and White individuals was 0.260 (95% CI, 0.227-0.298), indicating that, on average, racial equity in LDKT rates was not achieved at these centers, and more specifically, Black patients experienced inequity. The trajectory over time of LDKT rates tended to follow a cubic polynomial (eTable 2, eFigure 10 in Supplement 1), with evidence of improvement in LDKT rates for both Black and White patients in later years. Coverage of model estimations and 95% CIs was higher for White patients than Black patients, and model estimations more frequently overestimated the LDKT rates for Black patients relative to crude rates (eTable 3 in Supplement 1).

The standardized model coefficient estimates in Table 2 represent associations between each variable and the LDKT rates by race after adjusting for all other covariates. The percentage LDKT of total KT was associated with LDKT rates for both Black (coefficient, 0.356; SE, 0.027; *P* < .001) and White (coefficient, 0.304; SE, 0.014; *P* < .001) patients, and the coefficient was slightly higher for Black patients (coefficient, 0.052; SE, 0.03; *P* = .09). For White patients, there was a positive association between LDKT rate and interquintile range of ADI in TRRs (coefficient, 0.083; SE, 0.038; *P* = .03) and a negative association between LDKT rates and uninsured patients in TRRs (coefficient, –0.109; SE, 0.037; *P* = .003); these were not observed for Black patients (coefficient, –0.038; SE, 0.047; *P* = .42). Lack of Medicaid expansion was associated with a significantly greater decrease in LDKT rates for Black patients compared with White patients (coefficient, –0.161; SE, 0.068; *P* = .02). Race-specific percentage of patients with type B blood who were waitlisted was associated with LDKT rates for both Black (coefficient, 0.107; SE, 0.043; *P* = .01) and White (coefficient, –0.074; SE, 0.034; *P* = .03) patients, but in opposite directions; patients with type B blood were estimated to have fewer LDKTs if they were White, but more if they were Black. Race-specific percentage of patients with postsecondary education who were waitlisted was positively associated with LDKT rates for both and Black (coefficient, 0.095; SE, 0.038; *P* = .01) and White (coefficient, 0.185; SE, 0.029; *P* < .001) patients. Race-specific percentages of patients who were waitlisted with calculated panel-reactive antibody greater than 0.7 were positively associated with LDKT rates in Black patients (coefficient, 0.074; SE, 0.035; *P* = .03), but no such association was observed for White patients (coefficient, 0.033; SE, 0.027; *P* = .23).

**Table 2.** Model Coefficient Estimates and Coefficient of Inequity^a^

Effect	White			Black			Coefficient of inequity (Black minus White)		
	Coefficient	SE	*P* value	Coefficient	SE	*P* value	Coefficient	SE	*P* value
TRR characteristics									
No. of transplant centers in TRR	−0.018	0.036	.62	−0.006	0.042	.90	0.012	0.055	.83
Population-weighted interquintile range of ADI in TRR	0.083	0.038	.03	−0.013	0.044	.77	−0.096	0.058	.10
Population-weighted median percentage Black population in TRR	0.057	0.036	.11	0.005	0.042	.90	−0.052	0.055	.35
Population-weighted median percentage uninsured in TRR	−0.109	0.037	.003	−0.038	0.047	.42	0.071	0.06	.23
Center characteristics									
Medicaid expansion: no	0.05	0.029	.09	−0.111	0.061	.07	−0.161	0.068	.02
Paired exchange program: no	0.028	0.021	.19	0.005	0.044	.91	−0.023	0.049	.64
Percentage LDKT of total kidney transplant volume	0.304	0.014	<.001	0.356	0.027	<.001	0.052	0.03	.09
NKR voucher program: no	0.035	0.069	.61	−0.056	0.078	.47	−0.091	0.104	.38
Waiting list characteristics									
Percentage waitlisted patients with type B blood	−0.074	0.034	.03	0.107	0.043	.01	0.182	0.054	.001
Percentage female waitlisted patients	0.014	0.024	.57	−0.043	0.032	.18	−0.057	0.04	.16
Percentage waitlisted patients with some postsecondary education	0.185	0.029	<.001	0.095	0.038	.01	−0.09	0.048	.06
Percentage waitlisted patients with cPRA >0.7	0.033	0.027	.23	0.074	0.035	.03	0.041	0.044	.35

Abbreviations: ADI, Area Deprivation Index; cPRA, calculated panel reactive antibody; LDKT, living donor kidney transplant; NKR, National Kidney Registry; TRR, transplant referral region.

^a^ Standardized model coefficient estimates in the natural log scale of LDKT rates for Black patients, White patients, and the difference in the form of Black minus White adjusted for total wait days by race. The inequity coefficient of association represents the difference in regression coefficients (Black minus White). The *P* value refers to the test of the null hypothesis that the coefficient for the difference is equal to 0. Coefficients associated with numeric variables are standardized.

### Model-Based Estimations Based on Hypothetical Optimized Modifiable Covariates

**Figure 2.**
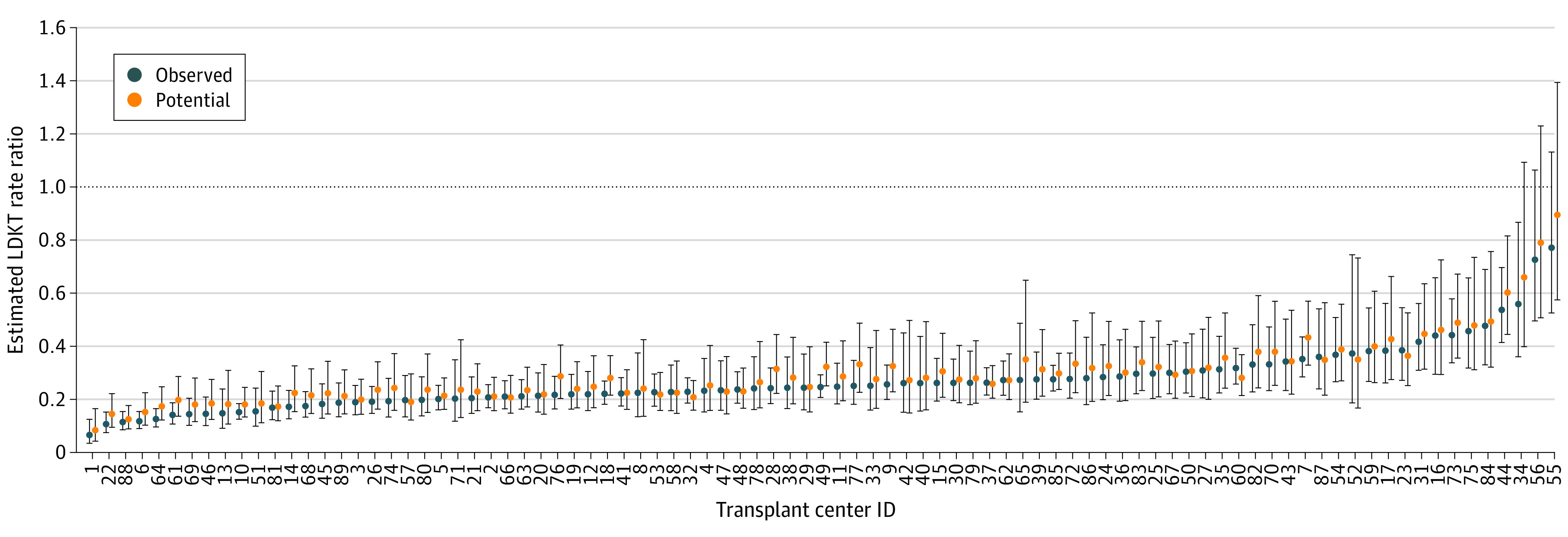
Estimated Living Donor Kidney Transplant (LDKT) Black-White Rate Ratios (RRs): Model Covariates Optimized for Equity Blue circles correspond to observed values of covariates; orange circles correspond to estimated LDKT RRs and associated 95% prediction intervals under a hypothetical scenario in which modifiable covariates for all transplant centers are fixed at values that promote equity of LDKT access while nonmodifiable covariates remain as observed. Error lines indicate 95% CIs. Note that the order of centers on the x-axis is identical to that of Figure 3.

Setting-modifiable center-level covariates to facilitate increased access to LDKT (participation in the NKR voucher program, participation in the paired exchange program, and state Medicaid expansion) resulted in a change in estimated minimum RR from 0.0557 to 0.0549 and an increase in estimated maximum RR from 0.771 to 0.895 (Figure 2; eFigure 8 in Supplement 1), implying improved equity. Relative to the observed 582 LDKTs in Black patients and 3837 LDKTs in White patients at 89 included centers in 2018, estimated total increase in LDKT volume would be 2261, of which 423 were additional for Black patients (a 72.7% increase) and 1838 were additional for White patients (a 47.9% increase).

### Model-Based Risk-Adjusted Estimations Based on Hypothetical Equalized Nonmodifiable Covariates

**Figure 3.**
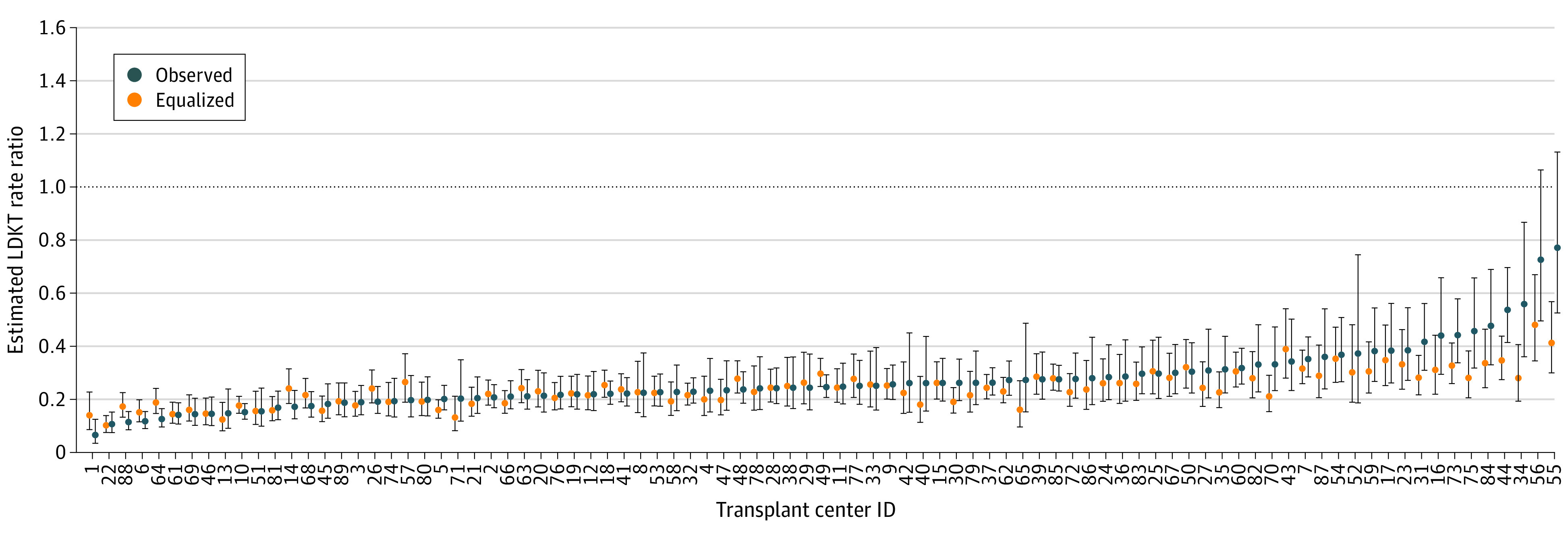
Estimated Living Donor Kidney Transplant (LDKT) Black-White Rate Ratios (RRs): Model Covariates Fixed at National Median Estimated LDKT RRs for all transplant centers in 2018. Blue circles correspond to observed values of covariates; orange circles to a hypothetical scenario in which nonmodifiable covariates for all transplant centers are fixed at their overall median values while modifiable covariates remain as observed. Error lines indicate 95% CIs. Note that the order of centers on the x-axis is identical to that of Figure 2.

Setting nonmodifiable covariates to their median values across all centers and all years while allowing modifiable factors to remain as they were observed resulted in an increase in the minimum risk-adjusted estimated RR between Black and White individuals from 0.0557 to 0.0878 and a decrease in the maximum risk-adjusted estimated RR from 0.771 to 0.515 (Figure 3; eFigure 9 in Supplement 1). Under this scenario, higher-performing centers were estimated to have reduced (less favorable) RRs between Black and White individuals while low-performing centers were estimated to have increased RRs between Black and White individuals.

## Discussion

In this study of US transplant centers from 2008 to 2018, we calculated yearly center-level RRs of LDKT rates for Black compared with White patients (where a RR of 1 indicates racial equity). Including patient, center, and regional characteristics, estimated LDKT RRs between Black and White individuals for center-years ranged from 0.0577 to 0.771 over the entire study period. No center consistently achieved estimated LDKT RRs between Black and White Individuals near 1. By-year medians of center-level–estimated LDKT RRs ranged from 0.216 to 0.285, indicating little improvement in the transplant system. Our findings are consistent with prior investigations reporting persistent racial disparities in LDKT over the past 3 decades.^5,9,40,41,42,43,44,45,46,47,48,49,50,51,52^ Our work builds on these studies by incorporating multilevel factors into an assessment of racial equity in LDKT and identifying modifiable factors that might serve as intervention targets. Characteristics associated with increased LDKT rates for Black patients were increased center volume of LDKTs compared with total KTs, higher patient educational levels, higher sensitization levels, higher race-specific type B blood, state Medicaid expansion, and prevalence of type B blood in the TRR.

Of those treatment options for kidney failure, LDKT has several advantages, including decreased time receiving dialysis, improved patient-reported quality of life, and superior long-term graft survival.^2,53,54^ Prior research has characterized patient-level barriers to racial equity in LDKT that include knowledge and attitudes toward LDKT, financial barriers, and risks of chronic kidney disease in the donor.^10,43,55,56,57,58,59^ Interventions aimed at overcoming patient-level barriers have been well received but are insufficient to increase LDKT rates in Black patients.^47,58,60,61,62,63,64,65,66,67^

Our findings suggest that national policy efforts are supportive of reducing LDKT inequities. The Advancing American Kidney Health Initiative introduced in 2019 and the Comprehensive Immunosuppressive Drug Coverage for Kidney Patients Act (HR 5534) are examples of national efforts that may reduce racial inequities by alleviating financial strain.^40,66,68,69^ Close monitoring of these policies and ongoing federal investigations into how to best reduce disincentives and barriers to living kidney donation will be essential to ensure they have their intended effect. Transplant clinicians and centers can also influence state health policy, as was observed with the Colorado change to the Medicaid payment rule allowing undocumented individuals with kidney failure to access maintenance dialysis.^25^

We additionally found that wider inequities in sociostructural deprivation within a TRR confers a benefit for White patients. The association between sociostructural inequity and LDKT rates between Black and White individuals is worrisome and suggests the need to evaluate other health and community-level factors that may contribute to LDKT racial disparities.^13,55^ This association may exist because transplant centers with the widest community-level disparity between the most and least deprived individuals have relatively fewer staff and tailored resources devoted to social support and transplant navigation, due to their ability to maintain clinical volumes and revenue from less disadvantaged, primarily White potential candidates.^70,71,72^ Further investigation is required to examine whether center-level incentives could ameliorate this association.

Our findings add to the growing body of literature associating community deprivation with access to and outcomes after organ transplant.^5,56,73,74,75,76,77,78,79^ Effective interventions to address these inequities will require expanding national data collection to include social determinants of health that are currently not available, both pretransplant (eg, at the level of referring nephrologists or dialysis clinicians) and at the transplant center level.^80^ The recent report from the National Academy of Sciences, Engineering, and Medicine^81^ sharpened the focus of these efforts, identifying the need to measure factors known to influence transplant evaluation, such as social risk assessments.^82,83^ In parallel, the United Network for Organ Sharing Kidney Paired Donation recently began a project to link third-party data to Organ Procurement & Transplantation Network in an effort to increase the depth and breadth of social determinants of health data available on transplant recipients. Regardless of the process, incorporation of reliable, longitudinal data that reflect the sociocontextual factors impacting transplant and living donor candidates is a requisite step to achieving racial equity in LDKT.

Center-level barriers contributing to transplant inequities have been acknowledged but not fully explored or incorporated into broader interventions.^13,44,54,84,85,86,87,88^ Continued progress in this area will require centers to examine their health systems and care processes through a health equity lens. This includes examining public-facing materials, tracking the prelisting transplant selection process and the pretransplant steps, implementing social worker and community health worker patient navigators to enhance patient- and community-level support, and adapting communication processes for patients with limited health and digital literacy and technologic access.^41,60,89,90,91^ Adoption of health equity measures into core quality processes such as quality assurance and performance improvement may facilitate centers identifying policies and protocols that have unintended consequences when imprecisely applied, such as *APOL1* testing and the living donor kidney evaluation algorithm, which embeds race into the equation.^92,93,94^ Participation in programs that facilitate LDKT for patients with incompatible donors, such as desensitization, NKR, and the United Network for Organ Sharing paired donation program, can reduce the burden of identifying potential donors for patients with reduced network size, but center participation is optional.^5,95,96,97,98,99^ It is likely that the degree of engagement in these programs has more of an impact than the binary option of in-or-out, and future work should examine how length and magnitude of exchange and donor benefit programs may improve equity in access to LDKT. Preliminary evidence also suggests that tailored programs should focus on expanding community-partnered approaches to increasing LDKT, including earlier engagement with dialysis facilities and nephrology practices.^60,100,101,102,103,104,105^

### Limitations

This study has several limitations, including those inherent to the use of national data registries, the discordant dates available for the TRR-level covariate data sources, and the derivation of the TRRs based on hospital referral regions. The unit of analysis was the transplant center; thus, the study did not account for competing risks for individual patients. The model covariates were chosen a priori, focus at the center and TRR levels, and do not account for unmeasured patient factors, such as the health of potential donors. In addition, while the range of ADI was accounted for as a covariate in our analysis, the magnitude of deprivation was not. The complexity and multitude of interactions and nuanced clinical judgments inherent in the transplant selection process and other care processes within the transplant center cannot be fully captured with currently available national data sources. As such, discerning which center-level disparities in access to transplant are true inequities is difficult due to differences in population sociodemographic factors across catchment areas, center processes, protocols for donor and recipient eligibility, and community resources. Patient-level variables in Scientific Registry of Transplant Recipients data, including calculated panel-reactive antibody, blood type, and educational level, all had missingness in less than 0.2% of observations in the current study. In addition, because cohort studies cannot establish causality, the associations identified should be interpreted in the context of the observable data. The use of registry data, which are designed to be a census of information—not a nonrandom sample—may eliminate some of the biases encountered in observational data analyses.

## Conclusions

The findings of this cohort study suggest that racial inequities in LDKT persist despite decades of investigation and intervention. Our findings observed geographic but no temporal variation and suggest that center participation in national programs, such as the paired exchange and voucher programs, may help to mitigate LDKT Black-White race inequities. Overall, our findings support the increasingly accepted notion that a strong program is multifactorial and many contributing factors remain unmeasured by national data systems. Achieving racial equity will require identification of LDKT RRs related to the referral region conditions, and tailored interventions and goal setting should be based on the center-specific barriers to achieve them.

## Notes

Supplement 1. **eMethods.** Detailed Methods **eResults.** Detailed Results **eTable 1.** Characteristics of Transplant Centers, Waitlisted Patients, and Referral Regions for All Study Years **eTable 2.** Fit Statistics for Models With Different Order Polynomial Random Effects Functions by Year **eTable 3.** Interval Coverage for Final Poisson Regression Model by Race in Terms of Percentage of Observed LDKT Rates That Fell Within the Model’s 95% Confidence Interval for the Predicted Value **eTable 4.** Mean Percent of Candidates Participating in UNOS KPD Program Match Runs Relative to Total Number of Live Donor Kidney Transplants Across All Centers in Each Year of the Study Period and Overall Across the Entire Study Period **eFigure 1.** CONSORT Diagram for Waitlisted Patient Cohort Used for LDKT Rate Denominators **eFigure 2**. CONSORT Diagram for Live Donor Kidney Transplants Used for Numerator of LDKT Rates **eFigure 3.** CONSORT Diagram for Transplant Center-Years Comprising Analysis Dataset **eFigure 4.** CONSORT Diagram for the Comprehensive Kidney Waitlist Cohort **eFigure 5.** CONSORT Diagram for the Cohort of Centers Listed as Currently Active by the Health Resources and Services Administration (HRSA Cohort) Used for Transplant Referral Region (TRR) Derivation **eFigure 6.** CONSORT Diagram for Comprehensive Kidney Transplant Cohort **eFigure 7.** Map of Transplant Referral Region (TRR) Catchment Areas Derived From Hospital Referral Regions (HRRs) Using Kidney Waitlist Population **eFigure 8.** Model-Based Predicted Live Donor Kidney Transplant (LDKT) Rate Ratios and Associated 95% Confidence Intervals Corresponding to a Hypothetical Scenario in Which Modifiable Covariates for All Transplant Centers Are Fixed at Values That Promote Equity of LDKT Access While Non-Modifiable Covariates Remain as Observed **eFigure 9.** Model-Based Risk-Adjusted Predicted Live Donor Kidney Transplant (LDKT) Rate Ratios and Associated 95% Confidence Intervals Corresponding to a Hypothetical Scenario in Which Non-Modifiable Covariates for All Transplant Centers Are Fixed at Their Overall Median Values While Modifiable Covariates Remain as Observed **eFigure 10.** Estimated Time Trend in Mean LDKT Rates for Each Race With 95% Confidence Bands
eReferences

Supplement 2.
Data Sharing Statement
