# Supplementary material for: Racial Equity in Living Donor Kidney Transplant Centers, 2008-2018
Source: JAMA Netw Open. 2023 Dec 15;6(12):e2347826. doi: 10.1001/jamanetworkopen.2023.47826 (PMC10724764; doi:10.1001/jamanetworkopen.2023.47826)
Supplement: Supplement 1. — eMethods. Detailed Methods eResults. Detailed Results eTable 1. Characteristics of Transplant Centers, Waitlisted Patients, and Referral Regions for All Study Years eTable 2. Fit Statistics for Models With Different Order Polynomial Random Effects Functions by Year eTable 3. Interval Coverage for Final Poisson Regression Model by Race in Terms of Percentage of Observed LDKT Rates That Fell Within the Model’s 95% Confidence Interval for the Predicted Value eTable 4. Mean Percent of Candidates Participating in UNOS KPD Program Match Runs Relative to Total Number of Live Donor Kidney Transplants Across All Centers in Each Year of the Study Period and Overall Across the Entire Study Period eFigure 1. CONSORT Diagram for Waitlisted Patient Cohort Used for LDKT Rate Denominators eFigure 2. CONSORT Diagram for Live Donor Kidney Transplants Used for Numerator of LDKT Rates eFigure 3. CONSORT Diagram for Transplant Center-Years Comprising Analysis Dataset eFigure 4. CONSORT Diagram for the Comprehensive Kidney Waitlist Cohort eFigure 5. CONSORT Diagram for the Cohort of Centers Listed as Currently Active by the Health Resources and Services Administration (HRSA Cohort) Used for Transplant Referral Region (TRR) Derivation eFigure 6. CONSORT Diagram for Comprehensive Kidney Transplant Cohort eFigure 7. Map of Transplant Referral Region (TRR) Catchment Areas Derived From Hospital Referral Regions (HRRs) Using Kidney Waitlist Population eFigure 8. Model-Based Predicted Live Donor Kidney Transplant (LDKT) Rate Ratios and Associated 95% Confidence Intervals Corresponding to a Hypothetical Scenario in Which Modifiable Covariates for All Transplant Centers Are Fixed at Values That Promote Equity of LDKT Access While Non-Modifiable Covariates Remain as Observed eFigure 9. Model-Based Risk-Adjusted Predicted Live Donor Kidney Transplant (LDKT) Rate Ratios and Associated 95% Confidence Intervals Corresponding to a Hypothetical Scenario in Which Non-Modifiable Covariates for All Transpl [file jamanetwopen-e2347826-s001.pdf]

Supplementary Online Content

McElroy LM, Schappe T, Mohottige D, et al. Racial equity in living donor kidney transplant centers, 2008-2018. *JAMA Netw Open*. 2023;6(12):e2347826. doi:10.1001/jamanetworkopen.2023.47826

eMethods. Detailed Methods

eResults. Detailed Results

eTable 1. Characteristics of Transplant Centers, Waitlisted Patients, and Referral Regions for All Study Years

eTable 2. Fit Statistics for Models With Different Order Polynomial Random Effects Functions by Year

eTable 3. Interval Coverage for Final Poisson Regression Model by Race in Terms of Percentage of Observed LDKT Rates That Fell Within the Model's 95% Confidence Interval for the Predicted Value

eTable 4. Mean Percent of Candidates Participating in UNOS KPD Program Match Runs Relative to Total Number of Live Donor Kidney Transplants Across All Centers in Each Year of the Study Period and Overall Across the Entire Study Period

eFigure 1. CONSORT Diagram for Waitlisted Patient Cohort Used for LDKT Rate Denominators

eFigure 2. CONSORT Diagram for Live Donor Kidney Transplants Used for Numerator of LDKT Rates

eFigure 3. CONSORT Diagram for Transplant Center-Years Comprising Analysis Dataset

eFigure 4. CONSORT Diagram for the Comprehensive Kidney Waitlist Cohort

eFigure 5. CONSORT Diagram for the Cohort of Centers Listed as Currently Active by the Health Resources and Services Administration (HRSA Cohort) Used for Transplant Referral Region (TRR) Derivation

eFigure 6. CONSORT Diagram for Comprehensive Kidney Transplant Cohort

eFigure 7. Map of Transplant Referral Region (TRR) Catchment Areas Derived From Hospital Referral Regions (HRRs) Using Kidney Waitlist Population

eFigure 8. Model-Based Predicted Live Donor Kidney Transplant (LDKT) Rate Ratios and Associated 95% Confidence Intervals Corresponding to a Hypothetical Scenario in Which Modifiable Covariates for All Transplant Centers Are Fixed at Values That Promote Equity of LDKT Access While Non-Modifiable Covariates Remain as Observed

eFigure 9. Model-Based Risk-Adjusted Predicted Live Donor Kidney Transplant (LDKT) Rate Ratios and Associated 95% Confidence Intervals Corresponding to a Hypothetical Scenario in Which Non-Modifiable Covariates for All Transplant Centers Are Fixed at Their Overall Median Values While Modifiable Covariates Remain as Observed

eFigure 10. Estimated Time Trend in Mean LDKT Rates for Each Race With 95% Confidence Bands

eReferences

This supplementary material has been provided by the authors to give readers additional information about their work.

© 2023 McElroy LM et al. *JAMA Network Open.*

eMethods. Detailed Methods

Study period and cohort definitions

The study period for all cohorts was defined as January 1, 2008, to December 31, 2018. Two separate analytic cohorts were defined that included only Black or White individuals: A live donor kidney transplant (LDKT) cohort and a kidney waitlist entry cohort. The LDKT cohort consisted of kidney transplants from the USRDS standard analysis file 'TX' that met the following criteria (Figure SDC1): 1) Occurred at a USRDS facility that performed at least one transplant during the study period, 2) was not missing USRDS patient identifier, 3) patient received a LDKT, 4) patient race listed as either Black or White, 5) transplant date occurred within the study interval, 6) patient was 18 years or older at time of transplant, and 7) center identifier was present in the USRDS-OTC cross-reference file.

The kidney waitlist analysis cohort consisted of kidney transplant waitlist entries sourced from USRDS standard analysis file 'WAITSEQ_KI' that met criteria 1, 2, and 4 above plus the following (Figure SDC2): 8) At least one day of wait time occurred during the study period, 9) at least one day of wait time occurred after the patient's 18th birthday, 10) the facility identifier was not missing, 11) the interval start date did not occur after the end date, and 12) the patient identifier was present in the USRDS PATIENTS data file. The USRDS 'WAITSEQ_KI' file has been cleaned in several ways by USRDS as stated in the USRDS Researcher Guide, including that "overlapping waiting periods at the same center were collapsed," "known transplant dates are used to truncate waiting periods at all centers at which a patient is currently listed," and "patients with inactive waiting periods were removed; the waiting list sequence lists only active periods."

In order to obtain detailed patient information, including date of birth, the waitlist cohort was merged with the patient dataset ('PATIENT' standard analysis file) by USRDS_ID. Waitlist intervals for patients

© 2023 McElroy LM et al. *JAMA Network Open.*

who turned 18 while waitlisted were left-truncated on their 18th birthday, and all waitlist intervals were right-truncated at a known patient death date. Waitlist intervals with end dates that were missing were assumed to represent patients who were active on the waitlist as of the date of dataset creation and were administratively censored on the last day of the study period. Waitlist intervals were manually created for patients who received a LDKT without any recorded waitlist interval, or a re-transplant but had no corresponding waitlist interval. To accurately capture LDKT and waitlist events over the study period, facilities that had used multiple USRDS identifiers over time were collapsed into four-letter organ transplant center (OTC) codes using both a prior-to-current provider identifier cross-reference and a provider identifier to four-letter OTC code cross-reference provided by USRDS. An additional inclusive LDKT cohort was created from the USRDS standard analysis file 'TX' using the same criteria as the LDKT cohort described above (Figure SDC3) except without criteria 4) and thus included LDKTs of patients who belong to all racial categories.

Primary Outcome Derivation

LDKT rates for Black and White patients were calculated separately for each transplant center during each calendar year in the study period using the two cohorts above. Race-specific LDKT rates were defined as the ratio of the number of LDKT events to the total number of patient wait days recorded as follows:

$$LDKT\ Rate_{j,k,t} = \frac{LDKT\ Events_{j,k,t}}{Wait\ Time_{j,k,t}} \qquad (1)$$

where $j = 1 \ldots J$ race groups, $k = 1 \ldots K$ transplant centers, and $t = 1 \ldots T$ calendar years in the study period. Multiple transplants received by a single patient in a given transplant center and year were each counted. Overlapping waitlist time for a single patient was counted when it occurred at separate centers but did not when it occurred for a single center. The waitlist entries were used to calculate the total time waited for each race at each center (i.e. aggregated by summation) and used as the denominator for the

LDKT rate outcome. In the case of multiple listings and a single transplant, the LDKT only counted for the center that performed it, while the wait time at all centers was counted towards each.

Crude Black-White LDKT rate ratios for each center k in each year t were calculated as the ratio of the Black LDKT rate to the White LDKT rate for each center in each study year:

$$LDKT\ Rate\ Ratio_{k,t} = \frac{African\ American\ LDKT\ Rate_{k,t}}{White\ LDKT\ Rate_{k,t}} \qquad (2)$$

In addition to the above metrics, total LDKTs were counted for each center in each year using the inclusive LDKT cohort described above. With these derived values, a cohort of organ transplant center-years (hereafter referred to as OTC-year cohort) was defined and the following inclusion criteria were applied: 1) At least 12 total LDKTs (irrespective of race) performed in all years of the study period, 2) LDKT rate was defined, and 3) OTC code was among those listed with GPS coordinates in the HRSA database. A total of 1,936 OTC-years were excluded by criterion 1 and one OTC-year for which the LDKT rate was undefined for Black patients as no wait time was recorded in 2017 was excluded by criterion 2.

<u>Covariates and Data Sources</u>

TRR-level

Demographic data from the 5-year estimates of the American Community Survey (ACS) for each of the years during the study period were acquired that included total, Black, and uninsured population estimates at census tract or block group spatial scales, as available.[1] In addition, Area Deprivation Index (ADI v3.0), which uses 2014-2018 5-year ACS estimates, was utilized to represent neighborhood adversity.[2,3] Specifically, weighted quintiles of ADI values within each TRR were calculated from the constituent block groups where the weight of each block group was proportional to the total population therein, and the inter-quintile range was derived for each TRR from these values. Census tract and block group polygons were obtained from the US Census TIGER/line shapefile database for both 2000 and 2010 decennial censuses.[4,5]

© 2023 McElroy LM et al. *JAMA Network Open.*

Center-level data

Center covariates consisted of percent LDKT of total kidney transplant volume, participation in the NKR voucher and UNOS paired exchange program, and state Medicaid expansion. UNOS provided the number of transplants performed through the Paired Exchange program for each transplant center in each year in the study period; participation in the program was defined as performing at least one transplant in a given calendar year. Participation in the National Kidney Registry (NKR) standard voucher program as of Jan. 12, 2023 for each transplant facility was obtained and applied retroactively to all years in the study period.[6] ACA expansion status for each state was obtained from the Kaiser Family Foundation website.[3]

Patient-level data

Patient data included PRA sensitization (cPRA), blood type, gender, and education attainment, and were obtained from the Scientific Registry of Transplant Recipients (SRTR) because they contained fewer missing values than the corresponding USRDS datasets. Specifically, blood type and education were sourced from SRTR data file 'CAN_KIPA' and cPRA from SRTR data file 'PRA_HIST'. For waitlisted patient variables, data were sourced from SRTR and missingness was very low (0.113% for cPRA, 0% for blood type, 0.171% for education). A SRTR-USRDS patient ID cross-reference was provided by USRDS.

<u>Statistical Model</u>

We modeled LDKT rates using a generalized linear mixed effects model in which a Poisson distribution and a log link function were assumed. The model included an offset term for log wait time and fixed effects for race, a cubic polynomial in time, and all covariates described above. Interactions with race were included for all other fixed effects, allowing for separate trends in LDKT rates to be fit for Black and White patients. The model also included a set of random effects for each race group within each

© 2023 McElroy LM et al. *JAMA Network Open.*

transplant center in order to account for covariance from repeated observations over time. The random effects took the form of a cubic polynomial over time and covariance among random terms was modeled as unstructured. This random effects structure was chosen because it achieved the optimal goodness-of-fit (BIC) and 95% prediction interval coverage compared to polynomials of other orders (Table SDC 2), and because it was not possible to include fully saturated random effects along with all covariates. While it is reasonable to expect that LDKT rates between Black and White patients within a given center and year would be similar and therefore correlated, we chose to model the covariance within centers among time explicitly because we expected that correlation to be stronger as patients typically wait at the same center across multiple years; unfortunately, it was not possible to account for both types of covariance during modeling. Model parameters were estimated using maximum likelihood estimation via adaptive quadrature using 'proc glimmix.'

Model validation

Over-dispersion for the final Poisson regression model was assessed in three ways: 1) The ratio of Pearson chi-square statistic to degrees of freedom was 0.78, 2) the estimate of the scale parameter in a version of the final regression model that assumes a negative binomial instead of Poisson likelihood was 0.000788, 3) density plots of predicted vs observed values were visually compared to density plots of random variates from a Poisson distribution. All methods validated the use of the Poisson likelihood. Interval coverage of the final model was examined by comparing observed LDKT rate values to the 95% intervals of model predicted values and is shown in Table SDC 3 below. Model diagnostic plots including scatterplots of conditional studentized residuals against linear predictors and of residuals against each covariate were examined.

Spatial dependence in the LDKT rate outcome could occur because centers that are closer geographically may share unmeasured similarities that would violate the assumption of independence of

© 2023 McElroy LM et al. *JAMA Network Open.*

the generalized linear mixed model utilized. To examine this possibility, empirical semi-variograms were generated by year and spatial correlation functions were fit separately for White and Black LDKT rates (the best-fitting among exponential, Gaussian, Matérn functions) using residuals from the final Poisson regression model via ‘proc variogram.’ Semi-variograms showed negligible spatial patterns in the residuals and statistical tests failed to reject the null hypothesis that the partial sill (scale term) was equal to zero, indicating no significant spatial dependence amongst the model residuals.

© 2023 McElroy LM et al. *JAMA Network Open.*

eResults. Detailed Results

Summary statistics related to LDKTs performed and characteristics of transplant centers, waitlisted patients, and referral regions are presented for all study years in SDC table 1.

Characteristics of Transplant Referral Regions

Annual population-weighted median percent of Blacks within TRRs ranged from 0 to 32.8% and population-weighted inter-quintile range of the ADI ranged from 7.7 to 53.4. Population-weighted median percent uninsured in referral regions ranged from 2.1% to 24.4%.

Center characteristics

A total of 89 included transplant centers performed between a minimum yearly count of 12 and maximum of 203 LDKTs over the 11-year study period. Annual kidney transplant volume ranged from 21 to 440 and annual LDKT ranged from 10 to 82% of overall kidney transplant volume. A total of 39.2% of centers participated in a paired exchanged program and 58.4% participated in the NKR voucher program. After 2010, between 50 (56.2%) and 60 (67.4%) centers were in states that benefited from state Medicaid expansion.

Characteristics of Waitlisted Patients

Waitlisted patients across all centers and years ranged from 0 to 100% female for Black patients and from 25.6 to 53.8 for White patients, percentage with some post-secondary education ranged from 0 to 100 for Black patients and from 7.95 to 72.1% for White patients, percentage with type B blood ranged from 0 to 40% for Black patients and from 3.7 to 18.1 for White patients, and percentage with cPRA > 70% ranged from 0 to 50 for Black patients and from 0 to 22.8 for white patients.

© 2023 McElroy LM et al. *JAMA Network Open.*

eTable 1. Characteristics of transplant centers, waitlisted patients, and referral regions for all study years

	2008		2013		2018		Overall	
	Black (N=89)	White (N=89)	Black (N=89)	White (N=89)	Black (N=89)	White (N=89)	Black (N=977)	White (N=978)
**Characteristics of Transplant Centers**								
**Number of LDKTs**								
Mean (SD)	47.5 (31.5)	47.5 (31.5)	50.9 (38.7)	50.9 (38.7)	54.9 (39.2)	54.9 (39.2)	50.3 (34.9)	50.3 (34.9)
Median [Min, Max]	36.0 [13.0, 167]	36.0 [13.0, 167]	35.0 [12.0, 187]	35.0 [12.0, 187]	39.0 [12.0, 199]	39.0 [12.0, 199]	37.0 [12.0, 203]	37.0 [12.0, 203]
**Percent LDKT**								
Mean (SD)	38.2 (13.4)	38.2 (13.4)	36.8 (14.7)	36.8 (14.7)	33.5 (13.1)	33.5 (13.1)	36.1 (13.4)	36.1 (13.3)
Median [Min, Max]	37.3 [11.6, 75.0]	37.3 [11.6, 75.0]	34.2 [10.0, 82.0]	34.2 [10.0, 82.0]	32.1 [12.2, 76.2]	32.1 [12.2, 76.2]	34.2 [10.0, 82.0]	34.2 [10.0, 82.0]
**Total kidney transplants**								
Mean (SD)	128 (68.9)	128 (68.9)	139 (78.2)	139 (78.2)	166 (87.9)	166 (87.9)	143 (77.6)	142 (77.6)
Median [Min, Max]	110 [32.0, 344]	110 [32.0, 344]	115 [22.0, 348]	115 [22.0, 348]	151 [21.0, 440]	151 [21.0, 440]	122 [21.0, 440]	122 [21.0, 440]
**Paired exchange program participation**								
No	89 (100%)	89 (100%)	42 (47.2%)	42 (47.2%)	49 (55.1%)	49 (55.1%)	594 (60.8%)	595 (60.8%)
Yes	0 (0%)	0 (0%)	47 (52.8%)	47 (52.8%)	40 (44.9%)	40 (44.9%)	383 (39.2%)	383 (39.2%)
**NKR voucher program participation**								
No	37 (41.6%)	37 (41.6%)	37 (41.6%)	37 (41.6%)	37 (41.6%)	37 (41.6%)	407 (41.7%)	407 (41.6%)
Yes	52 (58.4%)	52 (58.4%)	52 (58.4%)	52 (58.4%)	52 (58.4%)	52 (58.4%)	570 (58.3%)	571 (58.4%)
**ACA Medicaid expansion**								
No	89 (100%)	89 (100%)	89 (100%)	89 (100%)	29 (32.6%)	29 (32.6%)	690 (70.6%)	691 (70.7%)
Yes	0 (0%)	0 (0%)	0 (0%)	0 (0%)	60 (67.4%)	60 (67.4%)	287 (29.4%)	287 (29.3%)
**Characteristics of Waitlisted Patients**								
**Percent female waitlisted patients**								
Mean (SD)	41.5 (8.71)	39.4 (4.46)	40.2 (9.41)	38.4 (3.76)	38.9 (7.30)	36.4 (2.89)	40.4 (8.35)	38.2 (3.79)
Median [Min, Max]	42.6 [0, 60.0]	39.1 [25.6, 53.8]	41.4 [0, 60.5]	38.9 [27.5, 47.8]	39.6 [0, 63.2]	36.5 [29.8, 45.1]	41.7 [0, 100]	38.2 [25.6, 53.8]
**Percent waitlisted patients w/ some post-secondary education**								

© 2023 McElroy LM et al. *JAMA Network Open.*

Mean (SD)	46.4 (15.2)	48.9 (11.0)	51.3 (12.8)	51.5 (10.5)	58.7 (11.6)	55.3 (9.14)	52.5 (13.5)	52.2 (10.5)
Median [Min, Max]	46.6 [0, 85.0]	50.7 [7.95, 72.1]	50.8 [0, 84.3]	54.0 [12.9, 67.0]	58.3 [31.5, 100]	57.1 [33.7, 72.1]	52.3 [0, 100]	53.9 [7.95, 72.1]
**Percent waitlisted patients with type B blood**								
Mean (SD)	20.5 (6.14)	11.7 (2.26)	20.3 (5.20)	11.5 (2.46)	21.6 (5.02)	12.3 (2.21)	20.8 (4.95)	11.7 (2.27)
Median [Min, Max]	21.1 [0, 35.9]	11.6 [5.71, 17.5]	20.4 [0, 29.9]	11.3 [5.33, 18.1]	22.0 [0, 33.3]	11.9 [7.28, 17.4]	21.1 [0, 40.0]	11.7 [3.74, 18.1]
**Percent waitlisted patients with PRA > 0.7**								
Mean (SD)	3.35 (3.71)	3.42 (2.69)	10.7 (7.91)	9.00 (4.25)	10.8 (5.06)	8.44 (3.32)	9.03 (6.13)	7.68 (4.04)
Median [Min, Max]	2.70 [0, 25.0]	2.88 [0, 11.2]	9.89 [0, 50.0]	8.52 [1.52, 20.0]	10.3 [0, 28.6]	8.15 [2.25, 18.3]	8.57 [0, 50.0]	7.63 [0, 22.8]
**Characteristics of Referral Regions**								
**Pop-weighted inter-quintile range of Area Deprivation Index**								
Mean (SD)	37.1 (9.31)	37.1 (9.31)	37.1 (9.35)	37.1 (9.35)	37.1 (9.43)	37.1 (9.43)	37.1 (9.31)	37.1 (9.30)
Median [Min, Max]	38.5 [7.71, 52.3]	38.5 [7.71, 52.3]	38.2 [7.69, 52.5]	38.2 [7.69, 52.5]	38.2 [7.70, 53.4]	38.2 [7.70, 53.4]	38.3 [7.69, 53.4]	38.3 [7.69, 53.4]
**Pop-weighted median percentage African American population**								
Mean (SD)	6.09 (6.90)	6.09 (6.90)	5.64 (6.95)	5.64 (6.95)	6.06 (7.13)	6.06 (7.13)	6.05 (7.01)	6.04 (7.01)
Median [Min, Max]	2.71 [0.179, 27.0]	2.71 [0.179, 27.0]	2.35 [0, 28.9]	2.35 [0, 28.9]	2.68 [0, 32.4]	2.68 [0, 32.4]	2.70 [0, 32.8]	2.70 [0, 32.8]
**Pop-weighted median percentage uninsured**								
Mean (SD)	12.6 (4.67)	12.6 (4.67)	12.0 (4.57)	12.0 (4.57)	6.77 (3.38)	6.77 (3.38)	10.9 (4.73)	10.9 (4.73)
Median [Min, Max]	12.2 [3.71, 24.4]	12.2 [3.71, 24.4]	11.9 [3.34, 24.0]	11.9 [3.34, 24.0]	5.73 [2.10, 17.7]	5.73 [2.10, 17.7]	10.3 [2.10, 24.4]	10.3 [2.10, 24.4]
**Number of centers within TRR**								
Mean (SD)	1.85 (1.01)	1.85 (1.01)	1.85 (1.01)	1.85 (1.01)	1.85 (1.01)	1.85 (1.01)	1.86 (1.00)	1.85 (1.00)
Median [Min, Max]	2.00 [1.00, 4.00]	2.00 [1.00, 4.00]	2.00 [1.00, 4.00]	2.00 [1.00, 4.00]	2.00 [1.00, 4.00]	2.00 [1.00, 4.00]	2.00 [1.00, 4.00]	2.00 [1.00, 4.00]

The observational unit of the study was a race within a center for a single year. Common center-level characteristics were used for both races when modeling LDKT rates for a given center-year since both patient populations were waitlisted at that center in that year. TRR-level characteristics were drawn from the population as a whole in the TRR and were assigned to all centers located within that TRR. LDKT: living donor kidney transplant, NKR: national kidney registry, ACA: affordable care act, PRA: panel reactive antibody, TRR: transplant referral regions.

© 2023 McElroy LM et al. *JAMA Network Open.*

eTable 2. Fit statistics for models with different order polynomial random effects functions by year.

Fit statistic	Linear	Quadratic	Cubic	Quartic
-2 Log Likelihood	11,426.00	11,200.94	11,124.44	11,116.88
AIC (smaller is better)	11,488.00	11,272.94	11,208.44	11,214.88
AICC (smaller is better)	11,489.03	11,274.33	11,210.33	11,217.45
BIC (smaller is better)	11,586.64	11,387.49	11,342.07	11,370.78
CAIC (smaller is better)	11,617.64	11,423.49	11,384.07	11,419.78
HQIC (smaller is better)	11,528.00	11,319.39	11,262.63	11,278.10

© 2023 McElroy LM et al. *JAMA Network Open.*

eTable 3 Interval coverage for final Poisson regression model by race in terms of percentage of observed LDKT rates that fell within the model's 95% confidence interval for the predicted value. Columns indicating "under" denote percentages of observations for which the 95% confidence interval was entirely lower than the observed value, while columns indicating "over" denote the opposite scenario.

Black	White	Overall	Black under	White under	Black over	White over
51.9	76.3	64.1	18.5	10.4	29.6	13.3

© 2023 McElroy LM et al. *JAMA Network Open.*

eTable 4 Mean percent of candidates participating in UNOS KPD Program Match Runs relative to total number of live donor kidney transplants across all centers in each year of the study period and overall across the entire study period. Note that the first match run for the UNOS KPD Program was in October 2010.

Year	Mean % UNOS Paired Exchange
2008	0
2009	0
2010	1.47
2011	6.82
2012	8.81
2013	13.24
2014	14.88
2015	15.79
2016	14.21
2017	14.48
2018	13.66
Overall	9.39

© 2023 McElroy LM et al. *JAMA Network Open.*

eFigure 1. Consort diagram for waitlisted patient cohort used for LDKT rate denominators.

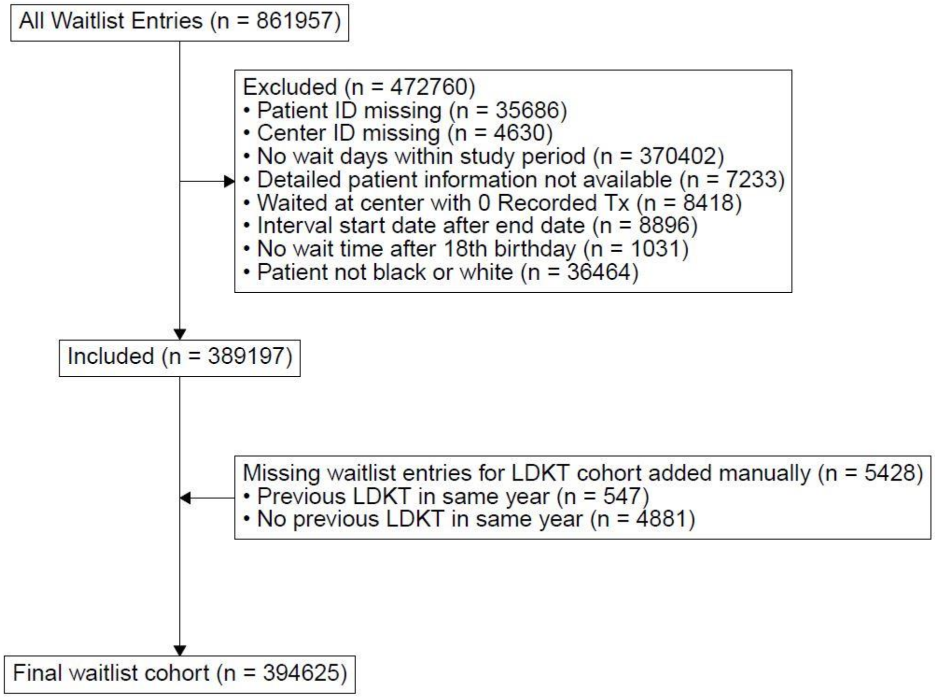

eFigure 2. Consort diagram for live donor kidney transplants used for numerator of LDKT rates.

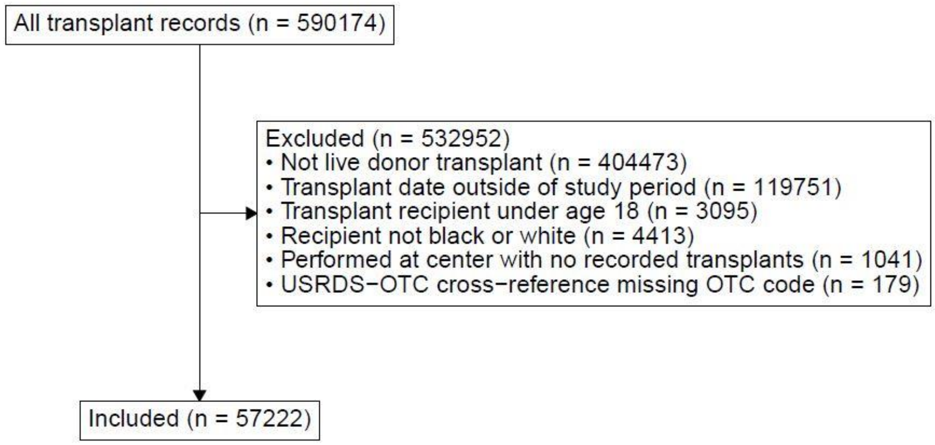

© 2023 McElroy LM et al. *JAMA Network Open.*

eFigure 3. Consort diagram for transplant center-years comprising analysis dataset.

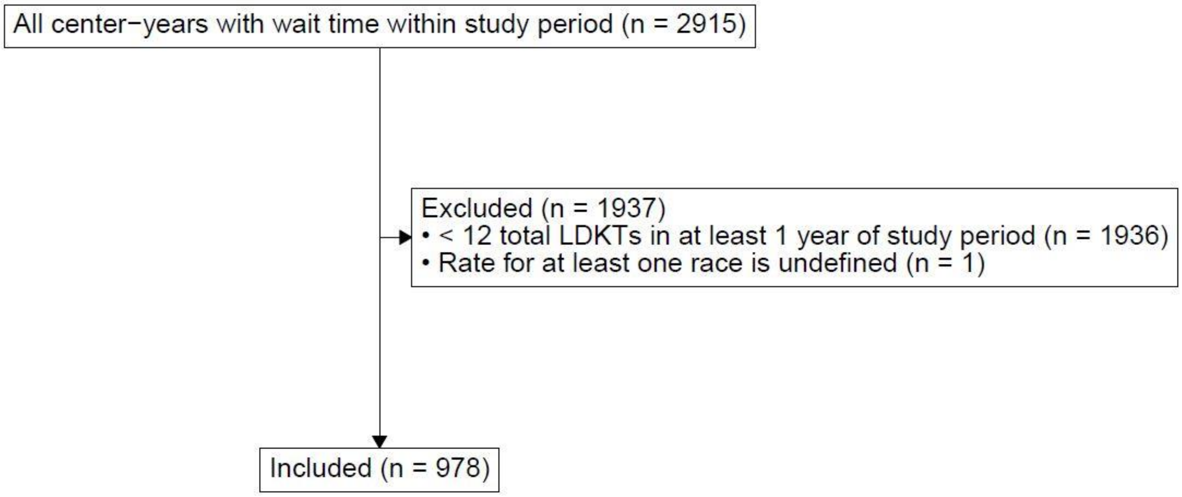

© 2023 McElroy LM et al. *JAMA Network Open.*

eFigure 4. Consort diagram for the comprehensive kidney waitlist cohort.

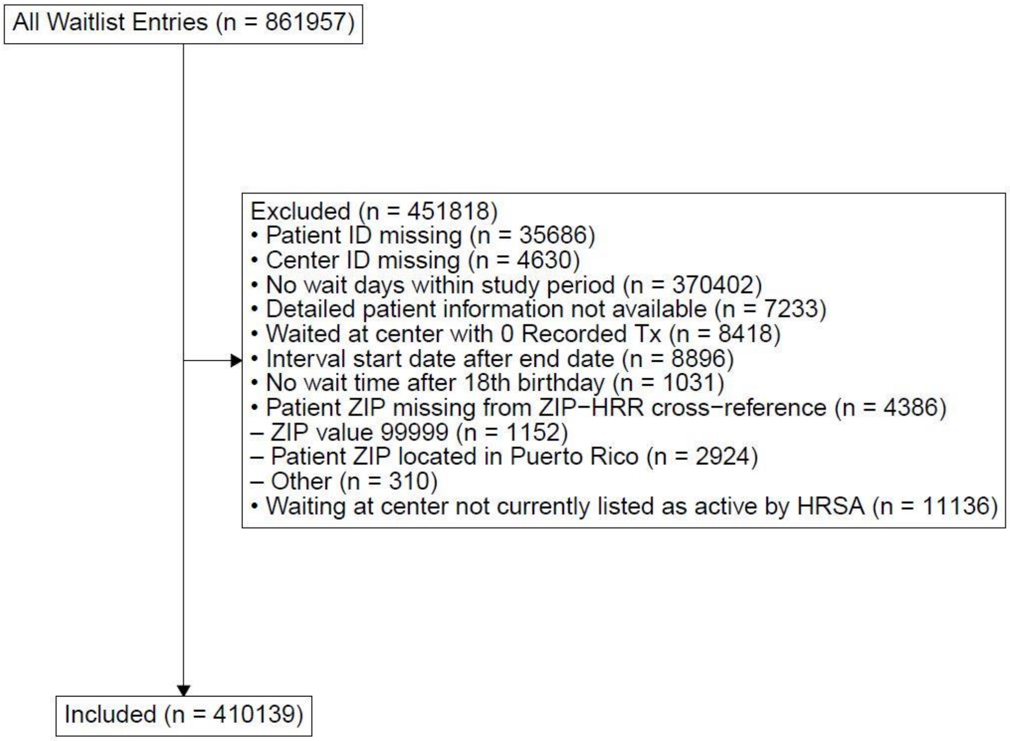

© 2023 McElroy LM et al. *JAMA Network Open.*

eFigure 5. Consort diagram for the cohort of centers listed as currently active by the Health Resources and Services Administration (HRSA cohort) used for transplant referral region (TRR) derivation.

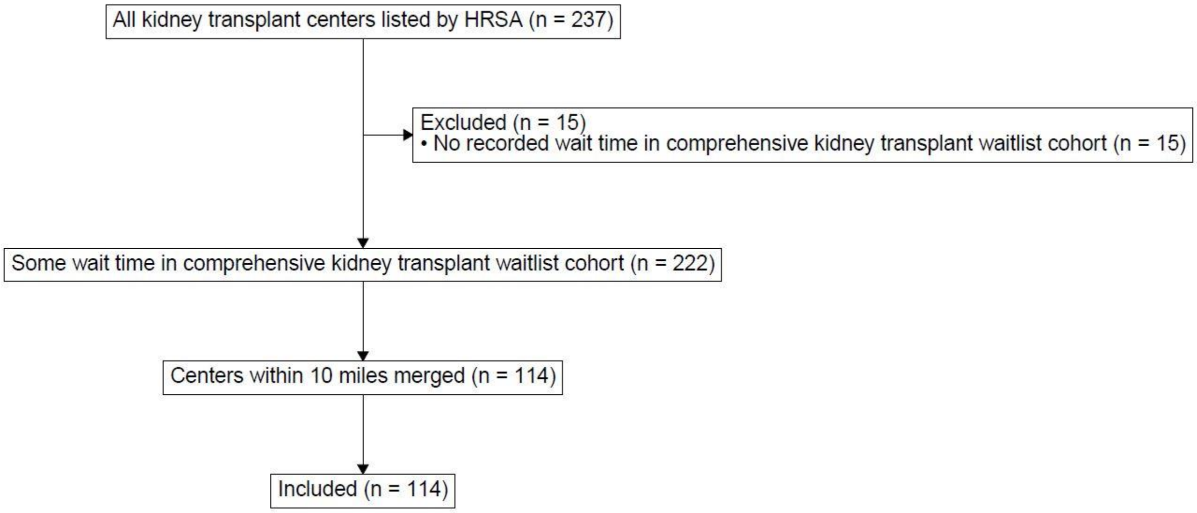

© 2023 McElroy LM et al. *JAMA Network Open.*

eFigure 6. Consort diagram for comprehensive kidney transplant cohort.

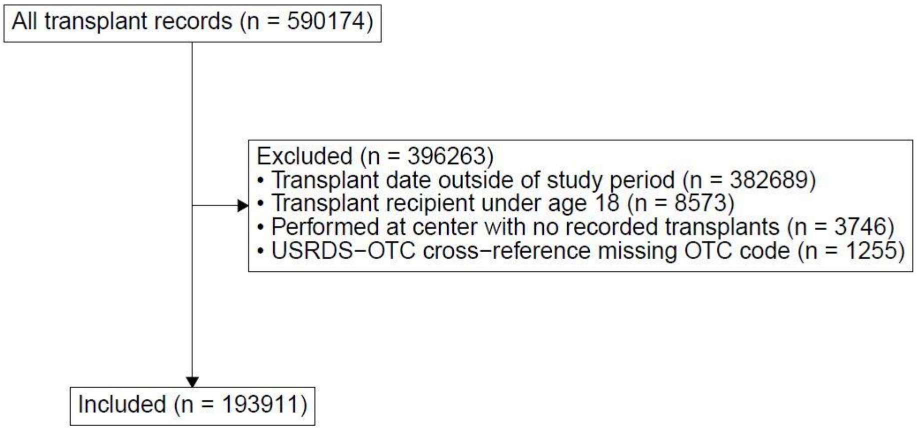

© 2023 McElroy LM et al. *JAMA Network Open.*

eFigure 7. Map of transplant referral region (TRR) catchment areas derived from hospital referral regions (HRRs) using kidney waitlist population.

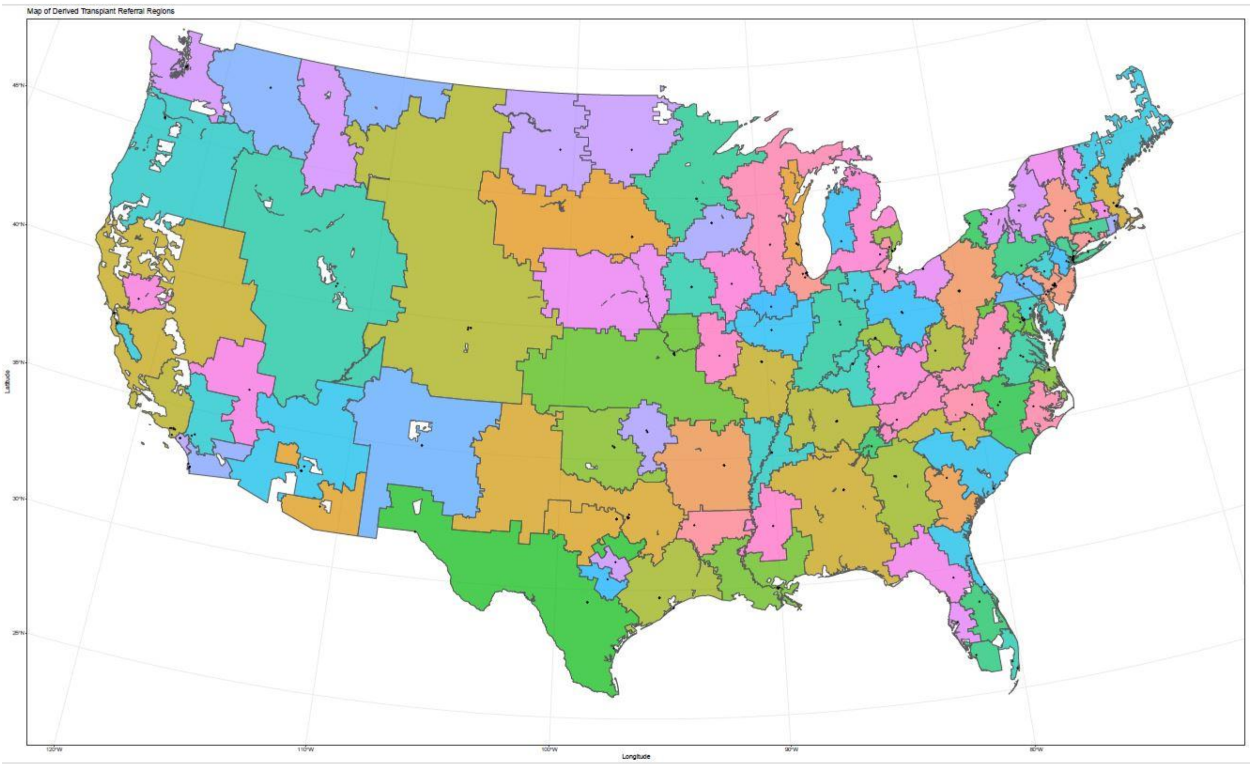

© 2023 McElroy LM et al. *JAMA Network Open.*

eFigure 8. Model-based predicted live donor kidney transplant (LDKT) rate ratios and associated 95% confidence intervals corresponding to a hypothetical scenario in which modifiable covariates for all transplant centers are fixed at values that promote equity of LDKT access while non-modifiable covariates remain as observed.

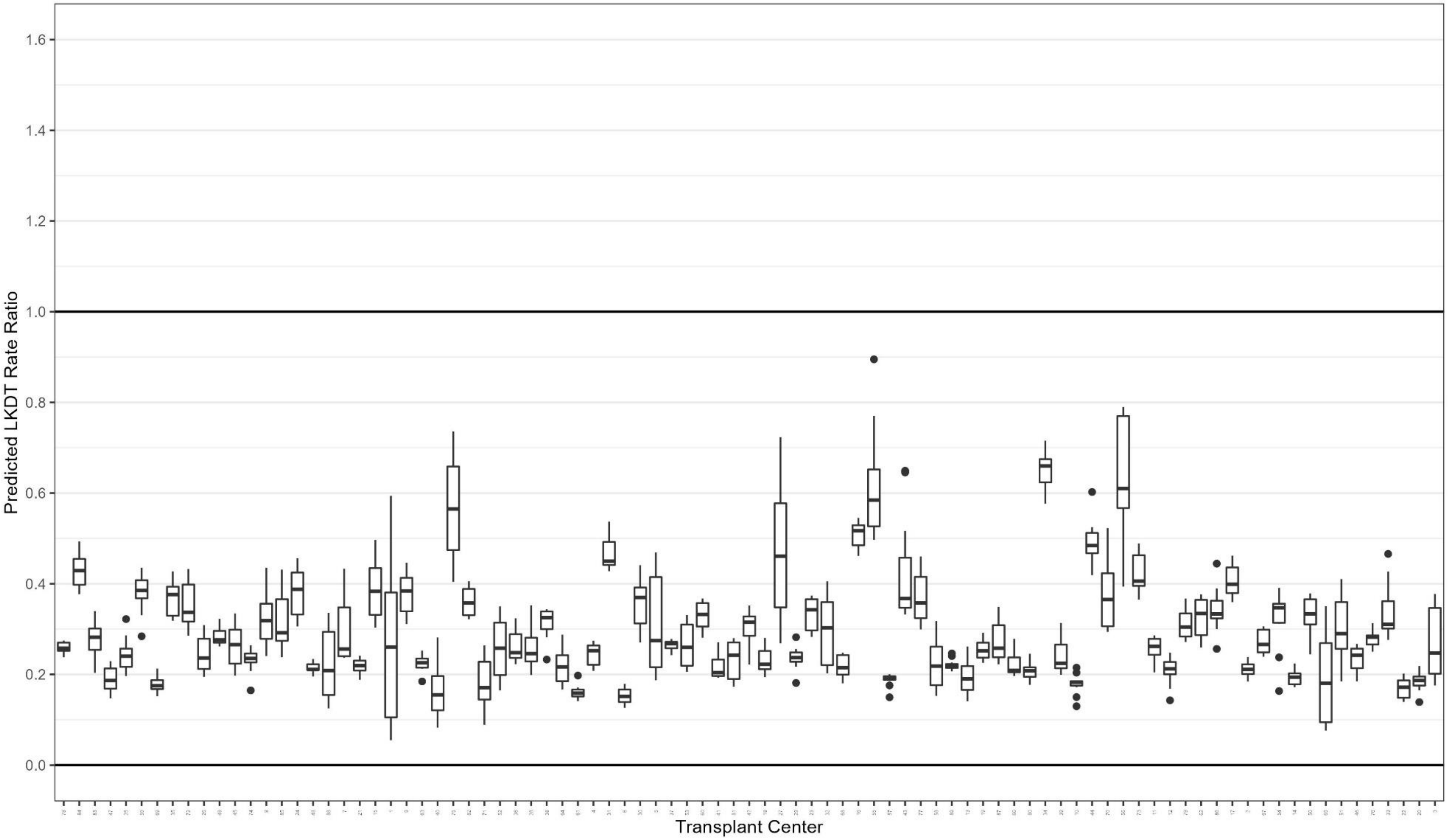

© 2023 McElroy LM et al. *JAMA Network Open.*

eFigure 9. Model-based risk-adjusted predicted live donor kidney transplant (LDKT) rate ratios and associated 95% confidence intervals corresponding to a hypothetical scenario in which non-modifiable covariates for all transplant centers are fixed at their overall median values while modifiable covariates remain as observed.

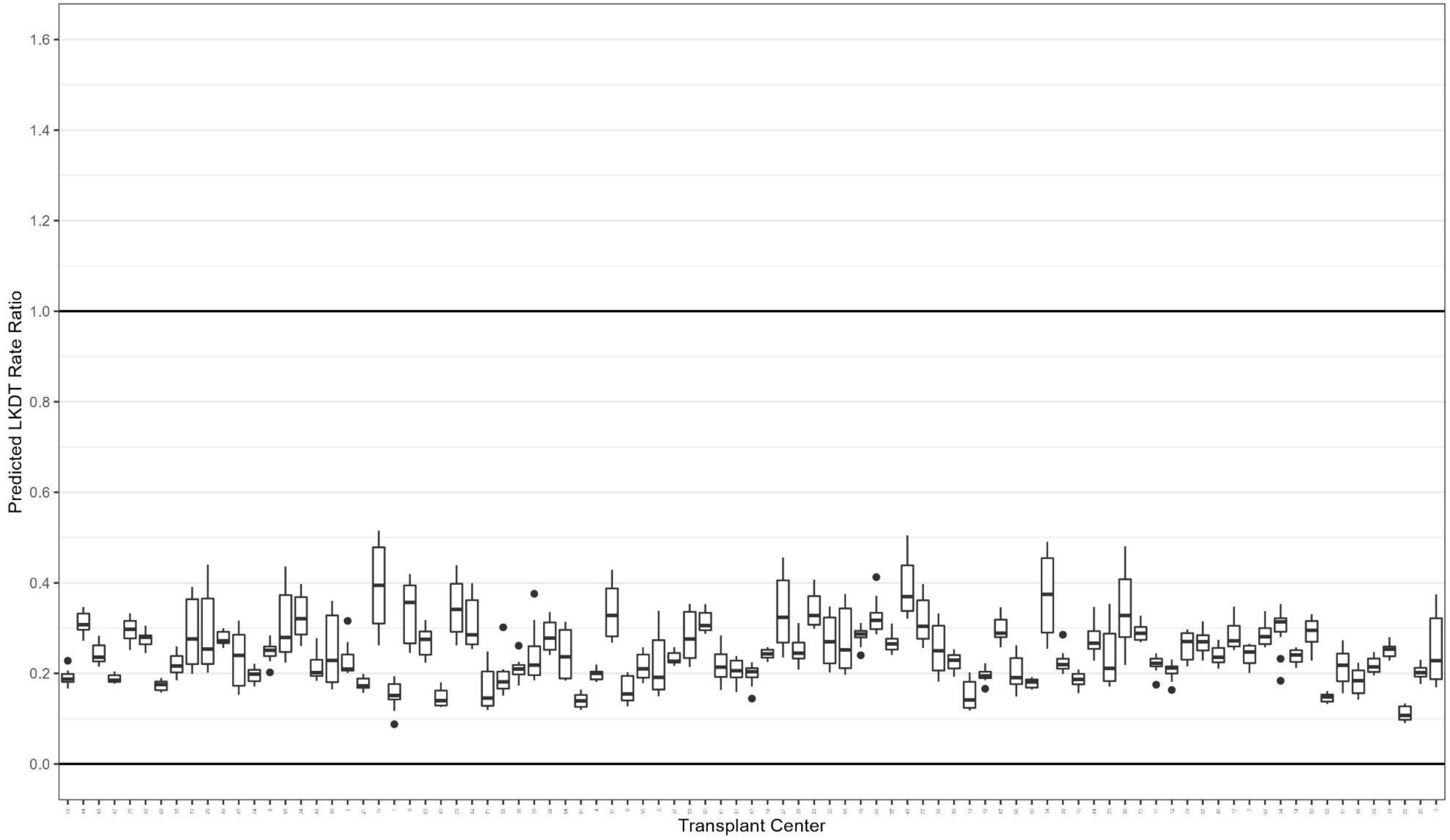

© 2023 McElroy LM et al. *JAMA Network Open.*

eFigure 10. Estimated time trend in mean LDKT rates for each race with 95% confidence bands. Dashed lines indicate yearly means of observed LDKT rates by race.

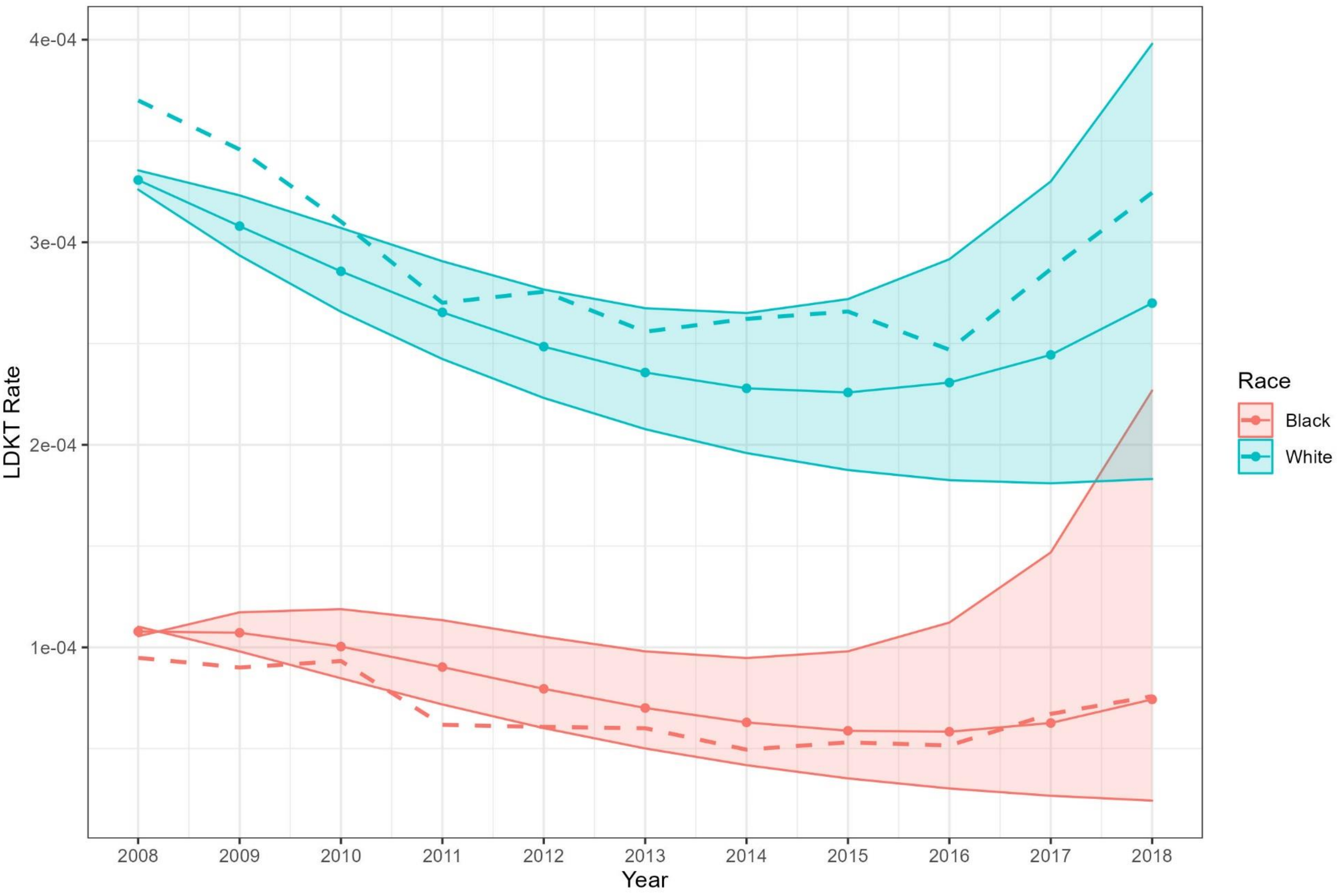

© 2023 McElroy LM et al. *JAMA Network Open.*

eReferences

1. Health Resources and Services Administration. Organ Donation and Transplantation. Updated 05/27/2021. Accessed Aug. 18, 2021. https://data.hrsa.gov//DataDownload/DD_Files/ORG_OTC_FCT_DET.xlsx
2. National Kidney Registry. Top Kidney Transplant Centers in the U.S. 2021. https://www.kidneytransplantcenters.org/
3. Foundation KF. Status of State Medicaid Expansion Decisions: Interactive Map. Updated 2022-02-24. Accessed Mar. 11, 2021. https://www.kff.org/medicaid/issue-brief/status-of-state-medicaid-expansion-decisions-interactive-map/
4. Kind AJH, Buckingham WR. Making Neighborhood-Disadvantage Metrics Accessible — The Neighborhood Atlas. *New England Journal of Medicine*. 2018-06-28 2018;378(26):2456-2458. doi:10.1056/NEJMp1802313
5. University of Wisconsin School of Medicine and Public Health. Area Deprivation Index (v3.0). 2018.
6. Tenenbaum EM. Swaps and Chains and Vouchers, Oh My!: Evaluating How Saving More Lives Impacts the Equitable Allocation of Live Donor Kidneys. *Am J Law Med*. Mar 2018;44(1):67-118. doi:10.1177/0098858818763812

© 2023 McElroy LM et al. *JAMA Network Open.*
