# Supplementary material for: Racial Equity in Living Donor Kidney Transplant Centers, 2008-2018
Source: JAMA Netw Open. 2023 Dec 15;6(12):e2347826. doi: 10.1001/jamanetworkopen.2023.47826 (PMC10724764; doi:10.1001/jamanetworkopen.2023.47826)
Supplement: Supplement 2. — Data Sharing Statement [file jamanetwopen-e2347826-s002.pdf]

Data Sharing Statement

Data
Data available: No

Additional Information
Explanation for why data not available: We have been prohibited from doing so by USRDS and UNOS
